# *In vivo* Imaging of Retina and Choroid in Guinea Pigs

**DOI:** 10.3389/fmed.2021.730494

**Published:** 2021-12-02

**Authors:** Li Dong, Yi Fan Li, Xue Jiang, Yin Jun Lan, Lei Shao, Jost B. Jonas, Wen Bin Wei

**Affiliations:** ^1^Beijing Key Laboratory of Intraocular Tumor Diagnosis and Treatment, Beijing Ophthalmology and Visual Sciences Key Lab, Medical Artificial Intelligence Research and Verification Laboratory of the Ministry of Industry and Information Technology, Beijing Tongren Eye Center, Beijing Tongren Hospital, Capital Medical University, Beijing, China; ^2^Department of Ophthalmology, Medical Faculty Mannheim, Heidelberg University, Mannheim, Germany; ^3^Institute of Molecular and Clinical Ophthalmology Basel, Basel, Switzerland; ^4^Privatpraxis Prof Jonas und Dr Panda-Jonas, Heidelberg, Germany

**Keywords:** *in vivo* imaging, OCT, retina, repeatability, reproducibility

## Abstract

**Purpose:** To evaluate the feasibility of *in-vivo* imaging of the retina and choroid using spectral domain optical coherence tomography (OCT) in guinea pigs.

**Methods:** The study included 19 pigmented guinea pigs (age: 3–4 weeks) which underwent sonographic axial length measurements and OCT imaging. At study end, the animals were sacrificed and histomorphometric examinations of the retina and choroid were performed. We assessed the reproducibility of the OCT measurements and compared *in-vivo* measurements to histomorphometric data.

**Results:** The mean thickness of the retina and choroid near the optic nerve head was 175.6 ± 25.8 and 63.4 ± 16.5 μm, respectively, and mean Bruch's membrane opening (BMO) diameter was 831 ± 121 μm. The intra-observer comparison of measurements of retinal thickness (intraclass correlation coefficient (ICC) = 0.92, 95% CI: 0.86–0.96; *P* < 0.001), choroidal thickness (ICC = 0.92, 95% CI: 0.86–0.96; *P* < 0.001), and BMO diameter (ICC = 0.92, 95% CI: 0.86–0.96; *P* < 0.001) showed a high correlation. A high agreement was present also for the inter-observer reproducibility of the measurements of retinal thickness (Pearson correlation coefficient (*R*) = 0.98; *P* < 0.001), choroidal thickness (*R* = 0.96; *P* < 0.001), and BMO diameter (*R* = 0.98; *P* < 0.001). The Bland-Altman plots showed that 2.6% (1/38), 5.3% (2/38), and 7.9% (3/38) of the measurement points of retinal thickness, choroidal thickness and BMO diameter, respectively, were located outside of the 95% limits of agreement. The OCT-based thickness measurements of retina and choroid were significantly higher than those measured by histomorphometry (both *P*-values <0.01).

**Conclusion:** OCT-based *in-vivo* morphometric imaging of the retina and choroid in guinea pigs is feasible with an acceptable intra-observer repeatability and inter-observer reproducibility.

## Introduction

Myopia has emerged as a common cause of vision loss as well as a major public health issue throughout the world, particularly in East Asia (1). It has been estimated that approximately half of the world population might be myopic by the year 2050, and 19.7% of the myopic individuals might be highly myopic (2). In a similar manner, a recent meta-analysis estimated that the prevalence of myopia in children and students aged 3–19 years in China might be higher than 80% by the year 2050 (3).

Axial myopia is characterized by the elongation of the ocular axial length. Previous studies have evaluated the morphometric changes of the sclera and choroid in axially elongated, myopic eyes of humans and animals (4–6). These investigations revealed that the thickness of the sclera and choroid progressively decreased with axial elongation, while the thickness of the retina in the macular region was independent of axial length (7–9). In some of the experimental studies, the measurements in animals were based on post-mortem histopathological examinations with the potential influence of confounding factors such as a post mortem swelling until the tissue was fixed by the fixative, and tissue shrinkage due to the processing of the histologic specimen. In addition, the histological method allowed the measurements taken only at one time point of the study, i.e., at the study end. It would therefore be advantageous to have the ability to measure the intraocular tissues in a non-invasive manner *in-vivo* by imaging techniques to monitor the morphometric changes during experimental studies addressing diseases such as myopia and glaucoma.

Spectral-domain optical coherence tomography (OCT) is a non-invasive imaging technique to visualize the posterior segment of the eye in humans and has markedly improved the clinical diagnosis of ocular diseases (10). OCT has also been used for rodent eyes to visualize the suprachoroidal space (11), assess corneal scarring (12), and detect vitreoretinopathy (13), to mention only few examples. Since the OCT technology allows the repeated intravital non-invasive imaging of the retina, retinal pigment epithelium (RPE), choroid and parts of, or the whole sclera, it has profound advantages as compared with the post mortem histomorphometric examinations of animals in experimental studies. Although numerous studies have addressed the repeatability and the inter-observer and intra-observer variation of the OCT measurements in humans, only few studies have assessed these parameters in animals included in experimental studies. It also holds true for investigations assessing the validity of the intravital OCT measurements by comparing the OCT figures with measurements obtained by histomorphometry. As an additional point, previous experimental investigations often used mice and rats or chicken while only recently, guinea pigs were increasingly used in studies, in particular in investigations examining the development and etiology of axial myopia (14–16). Guinea pigs as compared to chicken, mice and rats have the advantage of a larger eye size and an ocular anatomy relative similar to the human eye's anatomy (17). By the same token, most of the previous experimental studies were focused on the choroidal thickness, while the thickness of the retina was often not measured. Jnawali et al. (18) measured in guinea pigs the thickness of the retina as a whole and differentiated the retina into six retinal layers; the number of animals included in the study was however relatively low. We therefore performed the present study to further evaluate the feasibility of OCT imaging, with respect to its inter-observer and intra-observer variability and validity, for the non-invasive *in-vivo* measurement of the retina and choroid in normal young guinea pigs.

## Methods

The study included nineteen male pigmented Hartley Guinea Pigs (*Cavia porcellus*) with an age of 3–4 weeks and a mean body weight of 160.5 ± 10.5 g. All animals were obtained from the Fang Yuan-yuan farm, Beijing, China. The Ethics Committee of the Beijing Tongren Hospital approved this study. The ARVO Statement and ARRIVE Guidelines for the use of animals in ophthalmic and vision research were taken into account. All guinea pigs were kept in a quiet room with a constant temperature of 26°C. The light/dark cycle was 12h/12h with a light intensity of 500 Lux. All eyes included into the study were control animals and had not undergone any invasive procedure or lens-induced myopization.

Before the OCT examination, all animals were anesthetized with an intraperitoneal injection of 1% pentobarbital sodium (Catalog Number: 4579, R&D Systems, Bio-Techne Co., Minnesota, USA) with a dose of 4 mg pentobarbital/100 g body weight. The pupils of both eyes were dilated by mydriatic eye drops [2.5% tropicamide eye drops (Bauch and Lomb, NY, USA)]. A custom bracket was used to fix the animals and a custom lid speculum was applied to keep the eyelids open. Artificial tear eye drops (Hycosan, Ursapharm Arzneimittel GmbH, Saarbrücken, Germany) were topically applied to keep the cornea moist.

We measured the axial length of both eyes in each animal by ocular sonographic biometry (A/B-mode scan; oscillator frequency: 11 MHz; Quantel Co., Les Ulis, France) under topical anesthesia (oxybuprocaine hydrochloride eye drops; Santen Co., Osaka, Japan). The sound conducting velocity was set to be 1,557, 1,723 and 1,540 m/s for the measurement of the anterior chamber, lens and vitreous chamber, respectively (4). Ten measurements were taken for each eye, and the mean values were used for further statistical analysis. The standard deviation of the measurements had to be lower than 0.1 mm.

**Figure 1:**
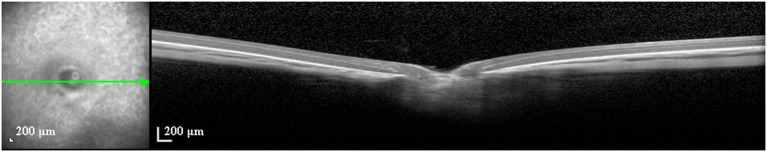
Unscaled image of an optical coherence tomographic image (enhanced depth imaging mode) through the optic nerve head of a guinea pig. Yellow lines: measurements of the thickness of retina and choroid, and of the diameter of Bruch's membrane opening.

**Figure 2:**
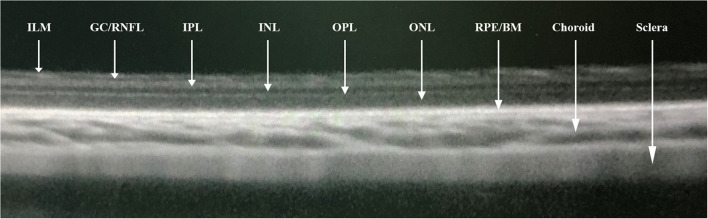
Segmentations of optical coherence tomographic image (enhanced depth imaging mode) showing the internal limiting membrane (ILM), ganglion cell/retinal nerve fiber layer (GC/RNFL), inner plexiform layer (IPL), inner nuclear layer (INL), outer plexiform layer (OPL), outer nuclear layer (ONL), retinal pigment epithelial/Bruch's membrane (RPE/BM), choroid, and sclera.

Each animal underwent OCT imaging of the retina and choroid (Spectralis, Wavelength: 870 nm; Heidelberg Engineering Co., Heidelberg, Germany) applying the enhanced depth imaging modality. Seven OCT sections, each comprising 100 averaged scans, were obtained with a scanned are a spanning 5° × 30°, which was centered on the optic nerve head. The horizontal section running through the center of the optic nerve head was selected for further analysis (Figure 1). The measurements of the OCT images were performed using the Heidelberg Eye Explorer software (version 5.3.3.0; Heidelberg Engineering Co.), as described previously (19). Both eyes of each animal were examined. If the quality of the scans was not sufficient, the OCT imaging was repeated. The different tissue layers in the OCT images were differentiated by their signal of reflection (Figure 2). The layers of the retina and choroid were defined and labeled as described in previous studies (18). The retina was stratified into the internal limiting membrane, ganglion cell/retinal nerve fiber layer, inner plexiform layer, inner nuclear layer, outer plexiform layer, outer nuclear layer, and RPE layer/ Bruch's membrane. The thickness of the choroid was measured as the distance from the hyperreflective line of the outer surface of the retina (i.e., the RPE line) to the hyperreflective line of the inner surface of the sclera. At the optic disc, the two end points of Bruch's membrane were manually marked. The distance between these two points were defined as the diameter of Bruch's membrane opening (BMO). In the horizontal sections running through the center of the optic nerve head, the region for the measurement of the retinal and choroidal thickness was located in a distance of one optic disc diameter temporal to the optic disc.

To assess the inter-observer variability, the scans were taken by one physician (YFL) and the images were measured in a masked manner by two trained ophthalmologists (LD, XJ). For the evaluation of the intra-observer variability, each image was measured twice at two different occasions by the same ophthalmologist (LD). To assess the inter-ocular differences, all measurements were calculated and compared between both eyes of each guinea pig.

At the end of the study, all animals were sacrificed by an intraperitoneal injection of an overdose of pentobarbital sodium (Catalog Number: 4579, R&D Systems, Bio-Techne Co., Minnesota, USA), followed by enucleation of both eyes. A total of ten eyes of ten animals were randomly selected for histopathological examination. The orbital fascia tissues were removed and the temporal position of the globe was marked. After fixation in a solution of 4% formaldehyde solution for 48 h, we prepared paraffin-embedded sections with a thickness of 8 μm and stained them with hematoxylin and eosin. Upon light microscopy, the sections containing the optic disc were selected for further histomorphometric assessment. The layers labeled in the OCT corresponded to the layers in the histopathological sections and the region for the measurement in both techniques was located at a distance of one optic disc diameter temporal to the optic disc. Using a digital image analysis software (Image-Pro Plus; Media Cybernetics Inc., Rockville, MD, USA), we measured the thickness of the choroid, the various layers of the retina, and the BMO diameter.

The statistical analysis was performed using a commercially available software program (SPSS 25.0; IBM Corp., Armonk, NY, USA). We calculated the mean ± standard deviation for continuous variables including body weight, axial length and tissue thickness measurements. Linear regression models were applied to analyze the associations of axial length and various *in-vivo* tissue thickness measurements. The intraclass correlation coefficient (ICC) with the 95% confidence interval (CI) was calculated for the intra-observer variability. A Bland-Altman analysis was performed for the assessment of the intra-observer consistency. The Pearson correlation coefficient (*R*) was calculated for the assessments of the inter-observer and inter-ocular measurements. Scatterplots were created to show the correlation between the measurements by two observers. The Student's *t*-test for paired samples was applied for the comparison of the *in-vivo* measurements and the histopathological measurements. A two-sided *P*-value <0.05 was considered to be statistically significant.

## Results

Nineteen pigmented guinea pigs (38 eyes) were included into the study. The mean axial length was 8.12 ± 0.07 mm. In the region of interest, the mean thickness of the choroid and retina were 63.4 ± 16.5 and 175.6 ± 25.8 μm, respectively. The mean BMO diameter measured 831 ± 121 μm.

The mean inter-eye difference in the measurements of the thickness of the choroid and retina (right eyes minus left eyes) was −4.05 μm (95% CI: −11.51, 3.41), and −22.76 μm (95% CI: −34.35, −11.18), respectively (Table 1). The mean inter-eye difference in BMO diameter (right eyes minus left eyes) was −51.99 μm (95% CI: −104.61, 0.64). Overall, the BMO diameter and the thickness of choroid and retina were highly significantly correlated between the right eyes and left eyes (all *R* > 0.50, all *P*-values <0.05).

**Table 1:** Interocular analysis showing the difference of measurements between the right eyes and left eyes in guinea pigs.

**Parameters**	**Right eyes**	**Left eyes**	**Difference**	**95% CI**	***t***	***P*-value**	**Pearson correlation coefficient (*r*)**	***P*-value for *r***
Mean axial length (mm)	8.12 ± 0.08	8.12 ± 0.05	0.00	−0.03, 0.02	−0.46	0.66	0.82	0.000
Mean retinal thickness (μm)	164.2 ± 17.2	187.0 ± 28.5	−22.76	−34.4, −11.2	−4.13	0.001	0.54	0.017
Mean choroidal thickness (μm)	61.3 ± 16.4	65.4 ± 17.2	−4.05	−11.5, 3.4	−1.14	0.27	0.57	0.011
Mean BMO diameter (μm)	804.7 ± 121.6	856.7 ± 119.0	−51.99	−104.6, 0.6	−2.08	0.053	0.59	0.008

*BMO, Bruch's membrane opening; CI, confidence interval*.

The intra-observer variability in the measurements was assessed by the same observer at two occasions (Table 2), with an ICC of 0.92 (95% CI: 0.84–0.96; *P* < 0.001) for the choroidal thickness measurements, an ICC of 0.92 (95% CI: 0.86–0.96; *P* < 0.001) for the retinal thickness determinations, and an ICC of 0.94 (95% CI: 0.89–0.97; *P* < 0.001) for the measurements of the BMO diameter.

**Table 2:** Intra-observer analysis showing the repeatability of measurements performed by the same observer in two different occasions.

**Parameters**	**Measurement 1**	**Measurement 2**	**Difference**	**95% CI**	**ICC**	**95% CI**	***P*-value**
Mean retinal thickness (μm)	177.50 ± 24.38	176.66 ± 27.90	0.84	−2.55, 4.24	0.92	0.86, 0.96	0.000
Mean choroidal thickness (μm)	62.87 ± 16.08	63.24 ± 17.38	−0.37	−2.66, 1.93	0.92	0.84, 0.96	0.000
Mean BMO diameter (μm)	808.58 ± 124.19	833.47 ± 121.06	−24.89	−36.08, −13.71	0.94	0.89, 0.97	0.000

*BMO, Bruch's membrane opening; ICC, intraclass correlation coefficient; CI, confidence interval*.

**Figure 3:**
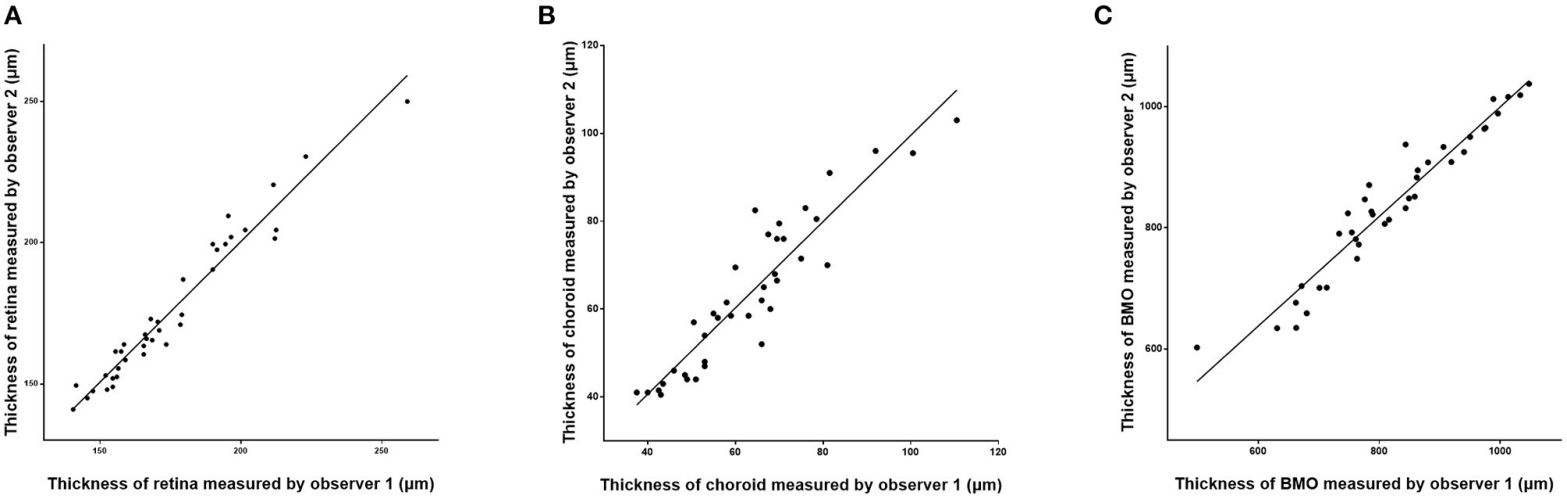
Scatter plots showing the correlation between the measurements of retinal thickness **(A)**, choroidal thickness **(B)**, and Bruch's membrane opening (BMO) diameter **(C)** measured on optical coherence tomographic images (enhanced depth imaging mode) by two independent observers.

**Figure 4:**
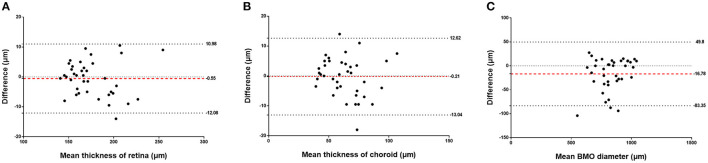
Bland-Altman analysis showing the inter-observer repeatability for measurements of the retinal thickness **(A)**, choroidal thickness **(B)**, and Bruch's membrane opening (BMO) diameter **(C)**. Red dotted line: mean bias, thick dotted lines: upper and lower limit of agreement, thin dotted line: baseline (zero).

The assessment of the inter-observer reproducibility revealed a high agreement between both examiners for the measurement of retinal thickness (*R* = 0.98; *P* < 0.001), choroidal thickness (*R* = 0.96; *P* < 0.001), and the BMO diameter (*R* = 0.98; *P* < 0.001) (Table 3; Figure 3). The Bland-Altman plots showed the percentage of measurement points located outside of the 95% limits of agreement was 2.6% (1/38), 5.3% (2/38), and 7.9% (3/38) for the measurement of retinal thickness, choroidal thickness, and BMO diameter, respectively (Figure 4).

**Table 3:** Inter-observer analysis showing the reproducibility of measurements performed by two observers independently.

**Parameters**	**Observer 1**	**Observer 2**	**Difference**	**95% CI**	**Pearson correlation coefficient (*R*)**	***P*-value**
Mean retinal thickness (μm)	174.05 ± 26.13	177.08 ± 25.68	−3.03	−4.62, −1.43	0.98	0.000
Mean choroidal thickness (μm)	63.68 ± 16.95	63.05 ± 16.38	0.63	−0.85, 2.11	0.96	0.000
Mean BMO diameter (μm)	840.38 ± 121.82	821.03 ± 121.45	19.36	12.09, 26.63	0.98	0.000

*BMO, Bruch's membrane opening; CI, confidence interval*.

**Figure 5:**
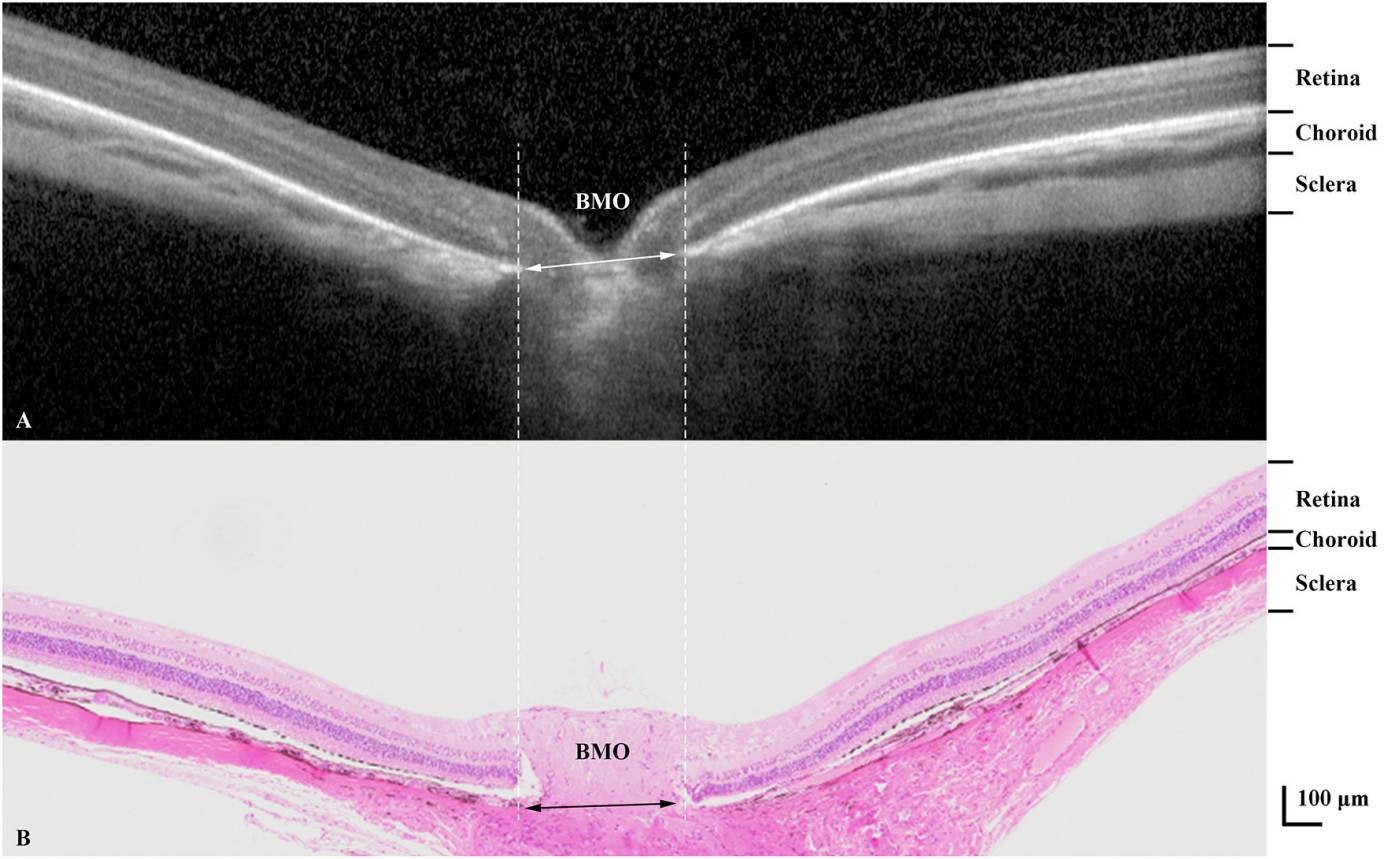
Optical coherence tomographic image (enhanced depth imaging mode) **(A)** showing various tissue layers with corresponding layers in the histological section **(B)**. BMO, Bruch's membrane opening.

Comparing the intravital OCT-based measurements with the histomorphometric determinations revealed higher values for the thickness of the choroid and retina as measured on the OCT images as compared to the values determined by histomorphometry (choroidal thickness: 69.00 ± 21.92 μm vs. 46.40 ± 15.09 μm, *P* = 0.002; retinal thickness: 175.05 ± 27.66 μm vs. 138.87 ± 7.10 μm, *P* = 0.001) (Table 4; Figure 5). The correlation between both the values obtained by the two measurement techniques was moderate (choroidal thickness measurements: *R* = 0.56, *P* = 0.09; retinal thickness measurements: *R* = 0.65, *P* = 0.04). Although the various retinal layers as seen on the OCT images corresponded to the retinal layers as detected on the histological slides, the tissue in the histologic slides showed an artificial tissue distortion and the retinas were often artificially detached from the RPE.

**Table 4:** Comparison of measurements performed by OCT and histomorphometry in 10 eyes of guinea pigs.

**Parameters**	**OCT**	**Histology**	**Difference**	**95% CI**	**t**	***P*-value**	**Pearson correlation coefficient (*R*)**	***P*-value for *R***
Mean retinal thickness (μm)	175.05 ± 27.66	138.87 ± 7.10	36.18	18.25, 54.12	4.56	0.001	0.56	0.093
Mean choroidal thickness (μm)	69.00 ± 21.92	46.40 ± 15.09	22.60	10.37, 34.83	4.18	0.002	0.65	0.044

*OCT, optical coherence tomography; CI, confidence interval*.

The mean thickness of the retina or choroid were not significantly correlated with axial length (both *R* < 0.30, both *P*-values > 0.05). In a similar manner, there was no significant relationship between body weight and the mean thickness of retina and choroid (both *R* < 0.40, both *P*-values > 0.10), nor between the BMO diameter and axial length or animal weight (both *R* < 0.20, both *P*-values > 0.40).

## Discussion

Assessing the feasibility of determining the thickness of the various retinal layers and of the choroid in guinea pigs *in-vivo* on OCT images showed that these measurements can be carried out with a relatively high intra-observer repeatability and inter-observer reproducibility. The examination of the validity of these OCT-based measurements by comparing the values with measurements obtained on histologic sections of the same eyes was markedly limited by artifacts in the histologic sections and artifacts inherent to any histologic preparation including the pre-fixation ischemic tissue swelling and the fixation-induced shrinkage of the tissue.

The stratification of the retina into eight layers as performed in our study what was similar to the differentiation of six retinal layers in a previous investigation by Jnawali et al. (18). In both studies, the point of measurement was located close to the posterior pole. The retinal thickness of 175.6 ± 25.8 μm as found in our study population was comparable to the retinal thickness measurements obtained in previous investigations also applying the OCT technique, with retinal thickness values of 142.2 ± 10.5 μm (measured at a distance of 3 mm from the optic nerve head) (20), 129 ± 1.3 μm (unspecified location) (21), and 151.2 ± 8.2 μm (total) (18). The choroidal thickness measurements assessed in the present study (63.4 ± 16.5 μm) were thinner than those obtained in previous studies with values of 120.9 ± 3.6 μm (20) and 134.2 ± 16.2 μm (21), while it was comparable with the results reported in the study by Jnawali et al. (69.8 ± 6.4 μm) (18). The differences in the choroidal thickness measurements may have been due to differences in the location of measurement.

The thickness of the retina and choroid as measured on the OCT images was higher than the histomorphometric determinations. It agrees with previous reports of studies on humans and animals. Jiao et al. (22) found that the tissue shrinkage due to the histologic processing differed between the retina and choroid. The choroidal thickness as measured in the paraffin-embedded histological sections was about half of that in OCT images (ratio in the present study: 0.67), while the histological retinal thickness was about 80% of that measured on the OCT images (ratio in the present study: 0.79). A similar result was obtained in an investigation on monkeys, and the concept of a “non-linear tissue shrinkage” was proposed (23). The basis for the histologic processing-associated non-linear tissue shrinkage as difference between the retinal tissue shrinkage and the choroidal tissue shrinkage may be due to differences in the composition of the tissues. Histopathological studies have shown that the shrinkage in the intercellular space is less marked than that in the intracellular compartment, with the intracellular shrinkage occurring mainly in the cytoplasm rather than in the cell nucleus (24). The shrinkage of compact tissue such as the retina with a relatively high nucleus-to-cytoplasm ratio may thus be less marked than the shrinkage of the choroid consisting predominantly of loose connective tissue with higher fluid content. An additional factor may be the loss of choroidal blood during and shortly after enucleation, before the enucleated globes were fixed.

The study conditions differed between Jnawali's study (18) and our study, with both investigator groups applying the same OCT device. Jnawali and colleagues used corneal contact lenses with a 3.5 mm central thickness on the cornea while we did not apply such lenses. This methodological difference might have led to differences in the optical system and differences in tissue thickness measurements. In addition, both studies varied in the age of the guinea pigs. Other investigators used different OCT devices. Lu et al. (21) assessed the eyes of guinea pig with lens-induced myopia with the Stratus OCT3 device (Carl Zeiss Co. Jena, Germany). Liba and associates (25) used the Optovue speckle-modulating OCT device (wavelength: 900 nm) to compare the cornea and retina between mice and human. Zhang et al. (16) applied an angio-OCT (Spectralis HRA + OCT; Heidelberg Engineering, Heidelberg, Germany) to detect changes in choroidal thickness and choroidal blood perfusion in myopic guinea pigs.

The thickness of the choroid was not related to axial length in our study population, although previous studies in humans and in guinea pigs showed an inverse correlation (7, 9, 26–28). The reasons for the discrepancy between the studies may be the small sample size in our study and that all animals of our study were healthy controls with major interindividual differences in axial length.

When the results of our study discussed, its limitations should be taken into account. First, the study might have been more valuable if the whole sclera could have been visualized and measured by OCT. However, we failed to reliably detect the outer border of sclera in the OCT examination so that the OCT-based thickness measurements of the sclera could not be taken for further analysis. Second, although the results showed a high intra-observer repeatability and inter-observer reproducibility in measuring the retinal and choroidal tissue thickness, the measurements need to be confirmed in a larger sample and by different examiners under routine working conditions. Third, anesthesia might have influenced the choroidal blood perfusion and thus the choroidal thickness. Fourth, only male guinea pigs were included into the study and the measurements were performed at only one time point (i.e., at an age of the guinea pigs of 3–4 weeks). Future studies may include also female animals and may carry out longitudinal examinations. Fifth, the study would have been strengthened if refractometry had been performed.

## Conclusion

In conclusion, our study shows the feasibility of OCT for *in-vivo* imaging of the retina and choroid in guinea pigs with acceptable intra-observer repeatability and inter-observer reproducibility. This technique provides a convenient and direct approach for repeated, non-invasive, and *in-vivo* imaging of the retina and choroid in guinea pigs and may be helpful for detecting retinal and choroidal changes due to disorders such as myopia in experimental studies with guinea pigs.

## Data Availability Statement

The data generated for this study are available on request to the corresponding author.

## Ethics Statement

The animal study was reviewed and approved by Beijing Tongren Hospital, Capital Medical University.

## Author Contributions

LD, YFL, XJ, YJL, LS, JJ, and WBW designed the study and contributed to manuscript revision and approved the submitted version. LD, YFL, XJ, YJL, LS, and WBW performed the measurements and generated the data. LD carried out the statistical analysis and wrote the first draft of the manuscript. All authors contributed to the article and approved the submitted version.

## Funding

This study was supported by the Capital Health Research and Development of Special (2020-1-2052); Science & Technology Project of Beijing Municipal Science & Technology Commission (Z181100001818003); the Beijing Municipal Administration of Hospitals' Ascent Plan (DFL20150201).

## Conflict of Interest

The authors declare that the research was conducted in the absence of any commercial or financial relationships that could be construed as a potential conflict of interest.

## Publisher's Note

All claims expressed in this article are solely those of the authors and do not necessarily represent those of their affiliated organizations, or those of the publisher, the editors and the reviewers. Any product that may be evaluated in this article, or claim that may be made by its manufacturer, is not guaranteed or endorsed by the publisher.
